# Damage Monitoring of Composite Adhesive Joint Integrity Using Conductivity and Fiber Bragg Grating

**DOI:** 10.3390/polym15061575

**Published:** 2023-03-22

**Authors:** Chow-Shing Shin, Liang-Wei Chen

**Affiliations:** Department of Mechanical Engineering, National Taiwan University, No. 1, Sec. 4, Roosevelt Road, Taipei 10617, Taiwan

**Keywords:** adhesive joint, fiber Bragg grating, carbon nanotubes, tensile failure, fatigue failure, full spectral response, structural health monitoring

## Abstract

Adhesive joints possess a number of advantages over traditional joining methods and are widely used in composite structures. Conventional non-destructive examination techniques do not readily reveal joint degradation before the formation of explicit defects. Embedded fiber Bragg grating (FBG) sensors and the resistance of carbon nanotube (CNT)-doped conductive joints have been proposed to monitor the structural integrity of adhesive joints. Both techniques will be employed and compared in the current work to monitor damage development in adhesive joints under tensile and cyclic fatigue loading. Most of the previous works took measurements under an applied load, which by itself will affect the monitoring signals without the presence of any damage. Moreover, most FBG works primarily relied on the peak shifting phenomenon for sensing. Degradation of adhesive and inter-facial defects will lead to non-uniform strain that may chirp the FBG spectrum, causing complications in the peak shifting measurement. In view of the above shortfalls, measurements are made at some low and fixed loads to preclude any unwanted effect due to the applied load. The whole FBG spectrum, instead of a single peak, will be used, and a quantitative parameter to describe spectrum changes is proposed for monitoring purposes. The extent of damage is revealed by a fluorescent penetrant and correlated with the monitoring signals. With these refined techniques, we hope to shed some light on the relative merits and limitations of the two techniques.

## 1. Introduction

Adhesively bonded joints have a number of advantages over traditional bolt or weld joints. They help to keep the structure surface smooth and avoid localized bearing load at stress-concentrating holes or material degradation due to localized heating. Adhesive joints significantly reduce stress by spreading load transfer over a large area, leading to better stiffness, better strength-to-weight ratios, and markedly reduced fatigue problems. In composite structures, they provide additional merits in helping to avoid fiber discontinuity [1,2] and possible edge delamination due to hole drilling. However, a severe weakness of adhesive joining is that it cannot be disassembled to inspect its integrity. On the other hand, service loading conditions such as impact, occasional overload, and long-term fluctuating loading may induce joint degradation/damages that lead to eventual structural failure. Some means to evaluate the structural health of an adhesive joint are required to ensure safe operation.

Non-destructive examination (NDE) techniques are available to detect defects in adhesive joints. These include ultrasonic techniques such as the traditional pulse-echo [3,4,5] or through transmission [4] methods, as well as the more advanced guided wave [6,7], acoustic microscopy [8,9], and electromagnetic acoustic transducer [10]. Non-ultrasound techniques such as electromechanical impedance spectroscopy using external [11] or embedded piezoelectric sensors [12,13,14], thermography [5,15,16], and shearography [4,17,18] have also been proposed. It has been pointed out that non-destructive inspection for defect techniques are mainly helpful at the fabrication stage for quality assurance and at the late stage of failure where explicit defects have formed but are ineffective for bonds weakened by degradation during the service stage [19,20]. In addition, NDE cannot reliably and readily detect kissing bonds, which are zero-volume fabrication defects with little or no strength [20].

Instead of directly looking for defects, there are techniques suitable for real-time monitoring of the integrity degradation of adhesive joints. These include strain/stiffness monitoring using back face strain gages [21,22,23], optical fiber sensors signal surveillance [24,25,26,27,28,29,30,31,32,33,34,35,36,37,38,39,40,41,42,43,44,45,46,47,48,49], and resistance monitoring of conductive adhesive joints [50,51,52,53,54,55,56,57,58,59,60,61,62,63,64,65,66,67]. Strain gages can only be applied to the outer surface. They will disrupt an otherwise smooth surface and are susceptible to environmental degradation. In addition, they may not possess sufficient fatigue life for long-term monitoring [68]. The resistance method is economical to deploy to large adhesive joints, but its applicability to adherends with high resistivity may be limited.

Optical fibers are known to have excellent fatigue endurance [68]. They can be embedded inside the bond to leave the external surface smooth, which is essential for aerodynamic structures. They are relatively free from environmental attack and have been used for general structural health monitoring [26,27,28]. There are different kinds of optical fiber sensors. In adhesive joints, both distributed sensing [29,30,31] and discrete fiber Bragg grating (FBG) sensors have been used [6,32,33,34,35,36,37,38,39,40,41,42,43,44,45,46,47]. The distributed sensors measure strain along the fiber and are equivalent to a train of strain gages, with the best available spatial resolution going down to the millimeter range. Discrete FBG sensors encode measured information in the wavelength of a characteristic spectrum. FBGs have often been treated as embedded strain gages to monitor the deformation of adhesive joints under mechanical load [32,33,34,35,36,37,38] and hygrothermal swelling [24,25,44,45]. This requires the FBG spectrum to be single-peaked. FBGs embedded in adhesive joints sometimes showed spectrum splitting on adhesive curing [24,44,47,48,49]. Moreover, applied loading and damages may cause heavy chirping [6,46,47]. It has been shown that if the deformation of the spectral shape instead of a single peak of the FBG is used, information regarding the initiation and development of degradation and damages may be gained [6,46,47]. However, in these works, changes in spectral shape relied on subjective visual judgment. A quantitative description of the spectral shape change may give a more objective evaluation and better sensitivity to small changes.

For resistance monitoring, the adhesive joints were made conductive by adding particles such as carbon black [50], graphene [51,52], and carbon nanotubes (CNTs) [53,54,55,56,57,58,59,60,61,62,63,64,65,66,67]. Owing to their extremely high aspect ratio, CNTs are especially suitable for forming a conductive network and so are used extensively in research works on the health monitoring of adhesive joints. CNTs are either dispersed into the adhesive [53,54,55,56,57,58] or prepared into a conductive sensing layer and embedded in the bond line [59,60,61,62,63,64,65,66,67]. The conductivity of the CNT-doped adhesive may come from direct contact between the CNTs [69,70] as well as the tunneling effect through neighboring tubes in close proximity [70,71,72]. When a CNT-doped adhesive joint is loaded, resistance changes may result from four different mechanisms: (1) change in the amount of direct contact of the overlapping nanotubes [69,70]; (2) change in the tunneling resistance resulting from changes in inter-tube distance between neighboring CNTs [70,71,72]; (3) the intrinsic piezoresistivity of individual CNTs due to strain-dependent energy band gap opening [73,74]; (4) reduction in conductive cross-sectional area due to debonding crack growth.

In demonstrating their health monitoring capability, some works employed the double cantilever bending specimen with a mode I crack in the adhesive joint [52,53,54,64,67]. Crack extension reduces the remaining ligament, and so resistance increases by mechanism (4) above. Other works [55,56,57,58,59,60,61,62,63,64,65,66,67] employed lap-shear joint specimens. This configuration is more relevant to the practical structural application of adhesive joints. They showed that on monotonically loading to failure, the resistance across the joint increases with applied load. All four mechanisms mentioned above have probably played some role in the observed increases in resistance. It should be noted that such resistance increases may not all be attributable to damages in the joint. Straining alone, without the occurrence of any damage, can increase the resistance through mechanisms (1)–(3). More nuanced differentiation between the effects of straining and damages is needed to obtain a clearer picture of the damage monitoring capability of CNT-doped adhesive joints. Moreover, the effect of different kinds of damage on the change in resistance needs to be clarified for the method to be of practical use.

Both the FBG sensor and the resistance method will be employed to monitor the occurrence and development of damages in adhesive joints, and their relative merits will be compared. In view of the shortfalls in the demonstrations of these methods on adhesive joints mentioned above, an attempt to quantify the FBG spectral shape change will be made to detect the onset and follow the development of damages. The resistance change due to joint damage will be separated from that due to straining. Supplementary liquid penetrant examination will also be applied to differentiate debonding and internal damages inside the joint. With these refined measuring procedures and techniques, the relative merits and limitations of the resistance monitoring and embedded FBG sensors in the monitoring of the structural integrity of adhesive joints will be compared.

## 2. Materials and Methods

### 2.1. Single Lap Joint Specimen and Conductivity Measurement

**Figure 1:**
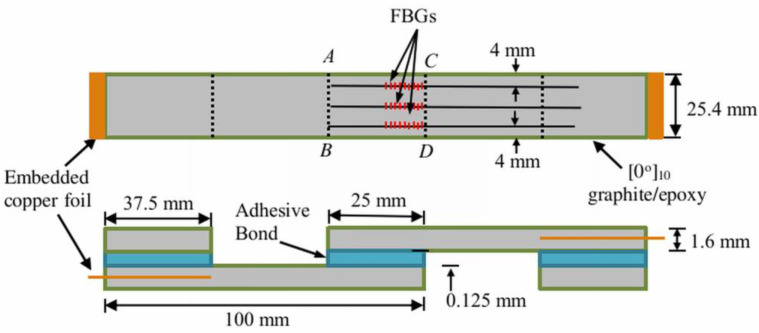
Dimensions and layout of the single lap joint specimen.

100 mm × 25.4 mm strips were cut from a 220 mm × 220 mm 10-ply uni-directional graphite-epoxy laminate. Single lap joint specimens with dimensions shown in Figure 1 were fabricated by gluing together two strips with conductive epoxy resin. The fiber direction in the composite is along the loading axis. Copper foil was pre-embedded at the end of the composite strips to allow for conductivity measurement. The areas to be joined were sanded, and masking tape was applied to the immediate vicinity beyond the boundary of the joint area. The purpose of the tape was to prevent excess glue from forming additional but unpredictable adhesion between the two parts. Excess glue is difficult to clean off thoroughly, especially with the optical fibers in place. Three optical fibers were embedded in the joint as spacers. The bond line was approximately as thick as the diameter of the optical fiber, i.e., 125 μm. In some of the specimens, there were FBGs in the optical fibers to act as sensors. The FBGs on the right, middle, and left sides are designated FBGL, FBGM, and FBGR, respectively. Stress analysis of single lap joint [75] indicated that for each of the stress components, stress concentration occurs right at or very close to the longitudinal edges of the joint (*AB* and *CD* in Figure 1). These will therefore be the most likely locations for initial damage and debonding to occur. Previous work [76] showed that an FBG along the loading direction straddling the longitudinal joint edge is more sensitive than other configurations in detecting joint damage. As the FBGs cannot cover the whole joint length, they were placed on one side of the joint so that their endpoints were slightly outside one longitudinal edge of the joint (see Figure 1) to stand a better chance of detecting damages.

Preliminary tests on the above fabrication procedures showed that the bond line has an even thickness and is free from gas bubbles. A section of the same composite strip was also glued to each end of the specimen to ensure the loading axis passed through the center of the adhesive layer.

A batch of seven single lap joint specimens can be made from a 220 mm × 220 mm composite laminate. Preliminary tests showed that their tensile strengths are affected by environmental conditions such as temperature and humidity during the joining operation. Different specimen batches had average batch strengths from 5.9 to 6.5 kN. However, within the same batch, the worst-case standard deviation of tensile strength was within 6.5%. For each batch, two specimens were tested under monotonic loading to failure to obtain the average batch tensile strength.

Conductive epoxy resin adhesive was prepared by mixing 0.3 wt% of amino-functionalized multi-walled carbon nanotubes (CNTs) in a room-temperature-cured Wah Hong WH-610S epoxy resin (Wah Hong Industrial Corp., Taipei, Taiwan). The CNTs (MWCNT-NH3-STD, Euflex Technology Corp., New Taipei City, Taiwan) have a nominal diameter of 9.5 nm and a nominal length of 1500 nm. To ensure good dispersion, the mixture was first sonicated with an ultrasonic homogenizer (UP200S, Hielscher Ultrasonics, Teltow, Germany) at 100 W for 5 min. This was followed by homogenization with a high-speed homogenizer (MiniBatch D-9, MICCRA, Heitersheim, Germany) at 21,000 rpm for 5 min. The mixture was buffered in iced water to cool down before the hardener was added and mixed thoroughly. The resulting mixture was degassed in a vacuum for one hour before application.

The change in conductivity of the adhesive joint was monitored via lead wires soldered to the pre-embedded copper foils at both ends of the lap joint specimen. A constant current was sent across the joint with a source meter (Keithley 2450, Tektronix, Inc., Beaverton, OR, USA), which also recorded the corresponding voltage drop simultaneously. The constant current employed was approximately 5 mA, and the initial voltage drop was approximately 3 V. This corresponds to an initial resistance of approximately 600 Ω. A 175 mm full-length composite strip without the joint has a resistance of approximately 3 Ω, so most of the voltage drop was across the adhesive joint. As the current was kept constant throughout a test, a change in voltage drop reflects a change in the resistance of the joint.

### 2.2. Fiber Bragg Grating Sensor

**Figure 2:**
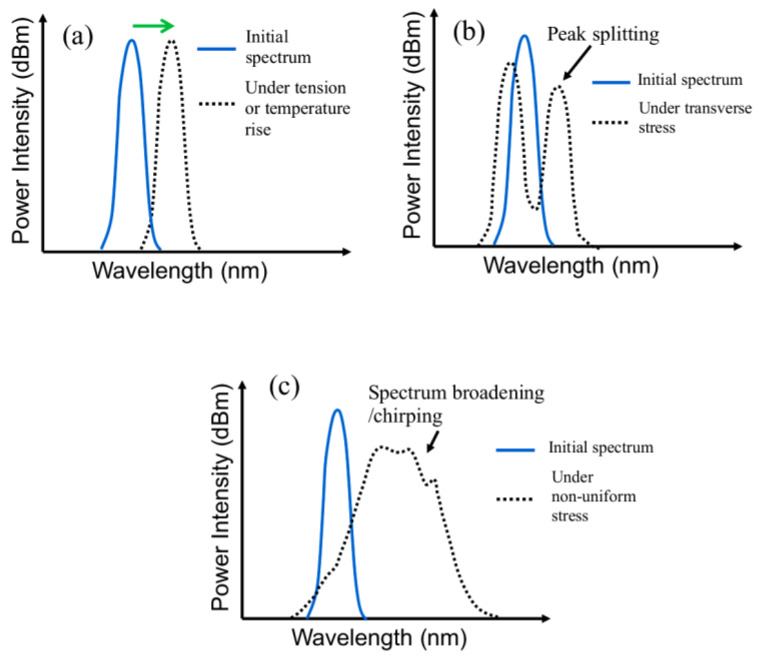
Schematic diagram to show FBG spectrum changes under (**a**) tensile stress or temperature rise, (**b**) transverse stress, and (**c**) non-uniform stress along its length.

A fiber Bragg grating (FBG) is a specific section on an optical fiber with a periodic variation of refractive index. With a uniform period Λ, the grating will reflect a characteristic single narrow peak spectrum with wavelength *λ* from an incident broadband light [77]:

*λ* = 2*n*Λ (1)

where *n* is the effective refractive index. When an FBG is subjected to a uniform longitudinal stress/strain, Λ will change due to the resulting strain and *n* will change by the photoelastic effect [77]. This causes a change in *λ*, and the reflected spectrum will shift as a whole, as shown schematically in Figure 2a. Typically for an FBG with *λ* = 1550 nm, a tensile strain of 1 με shifts the spectrum by approximately 1 pm towards the longer wavelength. Transverse stresses acting on the FBG will bring about a birefringence effect, resulting in a splitting of the spectrum peak (Figure 2b) [78]. If the stress/strain varies along the length of the FBG, different *λ*’s will satisfy the condition of Equation (1) and the spectrum will broaden or chirp, as shown schematically in Figure 2c. The shape of the chirped spectrum will be governed by the pattern of stress distribution.

Temperature change will change Λ and *n,* respectively, through thermal expansion/contraction and the thermo-optics effect [77]. For a freely standing FBG, this will lead to a shift of the whole reflected spectrum, such as that shown in Figure 2a. Typically for an FBG with λ = 1550 nm, a 1 °C rise shifts the spectrum by approximately 10 pm towards the longer wavelength. The above effects will superimpose if both temperature change and mechanical stress are applied to the FBG. When a compressive strain or a drop in temperature occurs, the spectrum shifts in the opposite direction towards the shorter wavelengths.

Curing of the adhesive involves localized temperature changes and volume contraction, leading to the occurrence of residual stress inside an adhesive joint. External load and temperature variation on the joint specimen will induce stress distribution inside the joint. When damages occur in the joint, they will act as local stress raisers. These will affect the load-induced stress distribution and cause a re-distribution of the residual stress. For an FBG embedded in a joint, the change of the FBG spectrum may therefore come from four contributions: (1) stress distribution in the joint caused by the applied load; (2) temperature change of the FBG; (3) residual stress distribution along the FBG; (4) perturbation of the stress distribution by damages. The resulting FBG spectrum will be affected by a superpositioning of all four effects. To reflect the effect of damage, the FBG spectrum is preferably measured under the load-free condition or at least under a minimal tensile load. A zero load does away with any contribution of applied load to the FBG spectrum. Any deviation of the instantaneous spectrum from the initial damage-free spectrum under the same load will indicate the occurrence and development of damages. A small tensile load contributes minimally towards the spectrum but may keep any damages open and enhance their perturbation effects.

In this work, uniform period FBGs were fabricated by side writing using a phase mask in a Ge-B co-doped single-mode optical fiber. The sensing length of the FBGs was approximately 10 mm, and the reflectivity of the as-produced FBG was approximately 99%. The reflected spectra from the FBGs were interrogated using an optical spectrum analyzer (MS9710C, Anritsu, Kanagawa, Japan). Mechanical testings were carried out in an air-conditioned room with thermostat control to within 1 °C, and the FBGs were embedded in poor thermal conductors (polymeric adhesive sandwiched between composite laminates) so that it is relatively insensitive to outside temperature changes. Small ambient temperature fluctuations will have a negligible effect on the measured spectra during mechanical testing.

### 2.3. Quantitative Comparison of FBG Spectra

As noted above, to discern the occurrence of internal damage, one should compare the instantaneous spectra with the initial reference spectrum under the same load to identify any change in shape or deformation. Visual comparison can only provide qualitative judgment. This is not preferable as it is subjective and may not be sensitive to discern minute changes. In a previous paper [79], a parameter was shown to be able to give an objective and quantitative assessment of the change in FBG spectra. This parameter, *D*, has been refined in the current work and is given by:

(2) $$D=100\sqrt{\frac{\sum_{i=1}^{m}{\left(p_{\lambda_{i}}^{current}-p_{\lambda_{i}}^{reference}\right)}^{2}}{m}}÷\delta$$

where $p_{\lambda_{i}}^{current}$ and $p_{\lambda_{i}}^{reference}$ are, respectively, the power intensity in dbm of the current unload spectrum and the reference spectrum at the same corresponding wavelength *λ_i_*; *δ* is a representative intensity of the reference spectrum and *m* is the number of data points in the wavelength span chosen to characterize the reference as well as the subsequently deformed spectra. For a series of spectra to be compared against an initial reference spectrum, the steps taken to calculate *D* are as follows: (1) the peak wavelength of each spectrum is shifted to overlap with that of the reference spectrum. This step aims to remove the effect of any temperature fluctuation that shifts the whole spectrum; (2) the starting and end point wavelengths of each FBG spectra are first identified based on the criterion that intensity changes at a rate greater than ±7 dbm/nm. Among the ranges between the starting and end points identified in different spectra, the smallest is noted; (3) to avoid irregularities near the background intensity, 70% of the range noted in step (2) is chosen to be the span for the calculation of *D*. The above two steps are to identify a common representative wavelength range throughout the whole degradation process for computation of *D*; (4) the representative intensity, *δ*, is calculated from the initial reference spectrum by computing the average power intensity within the chosen span minus baseline intensity, which is the average power intensities of the starting and end points of the reference spectrum. As the spectra from different embedded FBGs will differ from one another, normalization with *δ* makes the *D* values comparable across different FBGs.

### 2.4. Mechanical Testing

Insulating end tabs were bonded to the gripped area of the specimens. Specimens were subjected to tensile or cyclic loading on a servo-hydraulic testing machine (810 Materials Testing System, MTS Systems, Eden Prairie, MN, USA). Before testing, the initial reference FBG spectra were measured under 0 N and 400 N.

During the tensile test, loading was periodically interrupted to allow the reflected light spectra from the FBGs to be recorded at the instantaneous loading. The specimen was then unloaded to allow the spectra to be measured at 400 N and 0 N. This loading–unloading cycle was repeated with progressively higher loads until the specimen failed.

Fatigue testing of the specimens was carried out with a sinusoidal cyclic loading of 8 Hz. The cyclic loading range is 6–60% of the corresponding average batch tensile strength. Again, the tests were interrupted periodically, and specimens unloaded, to allow the FBG spectra to be measured at 400 N and 0 N.

### 2.5. Liquid Fluorescent Penetrant Treatment

As pointed out previously, the most critically stressed and most likely initial debonding regions are at the longitudinal edges of the joint. In order to show the instantaneous extent of debonding of the adhesive joint, some of the tensile and fatigue tests were interrupted before specimen failure. The specimens were then soaked in a fluorescent penetrant (Met-l-Chek FP-923, McGean, Cleveland, OH, USA). Soaking was carried out under zero load for 10 min, followed by a tensile loading of 50 N for 20 min, to facilitate the ingress of the penetrant into defects. The specimens were then wiped dry and cleaned superficially with a penetrant remover (Met-l-Chek E-59, McGean, USA). Afterward, the specimens were baked at 90 °C under a relative humidity of 30% in an environmental chamber (Model TTH-B1TP-S, Ten Billion Inc., Tainan, Taiwan) for 36 h. Preliminary tests showed that this duration and conditions could thoroughly dry the penetrant in a crevice formed by stacking together two pieces of the composite strip. After drying, the specimens were pulled to failure. The fracture surfaces were then observed under a 365 nm ultraviolet light to expose the extent of the dried penetrant.

## 3. Results

### 3.1. Monitoring of Tensile Damages

#### 3.1.1. FBG Spectrum under Progressively Loading Up-Unloading Cycle

**Figure 3:**
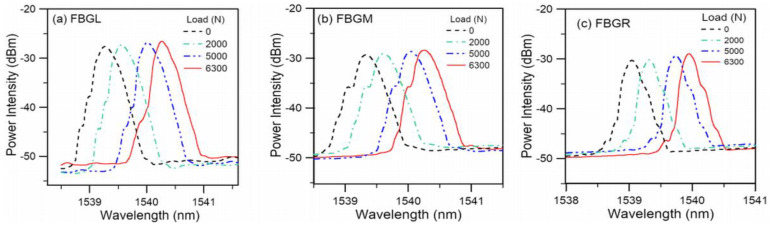
FBG spectra at various loadings in a typical tensile specimen.

Figure 3 shows the spectra from the three FBGs in a typical tensile specimen under different loading. With progressively increasing load, the spectra shift towards longer wavelengths. Only slight changes in the spectrum shapes are evident up to 6300 N. This was the load after which the last spectra were recorded before failure and corresponding to approximately 95% of the failure load.

**Figure 4:**
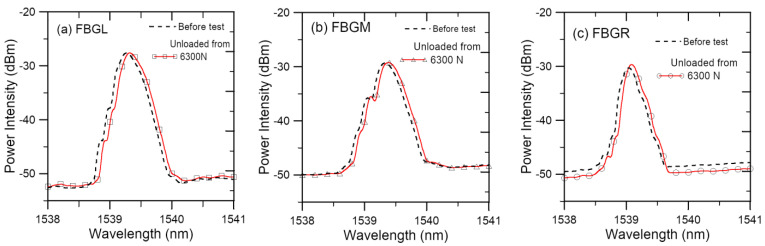
Load-free spectra measured after progressively loading up to 6300 N (~95% of failure load) in a typical tensile specimen.

To isolate any contribution of joint damages from that of applied load on the spectral shapes, load-free FBG spectra were measured on unloading after progressively loading up and are shown in Figure 4. Only the initial spectra and the last spectra measured before tensile failure are shown for clarity. The intermediate spectra during the course of loading lay between these two extremes and changed very little. In each of the spectra from the three FBGs, a slight shift to the longer wavelength is evident. However, the shape of the waveforms remained nearly unchanged. This trend was observed in 21 out of 24 FBGs. In these FBGs, not only the fully unloaded spectra but also the spectra measured on unloading to 400 N exhibited the same phenomena. Of the remaining three FBGs, one showed a progressively deformed waveform and the other two showed increasing background intensity, which gradually masked the peak. A shift in the peak wavelength with an unchanged waveform shape suggests that some uniform permanent tensile deformation has occurred, while the original strain distribution along the FBG has not been disturbed. The latter implies that any internal damages, if occurred, have not been serious enough to cause local stress concentration to perturb the strain field along the FBG.

#### 3.1.2. Conductivity Changes under Progressive Loading Up-Unloading

**Figure 5:**
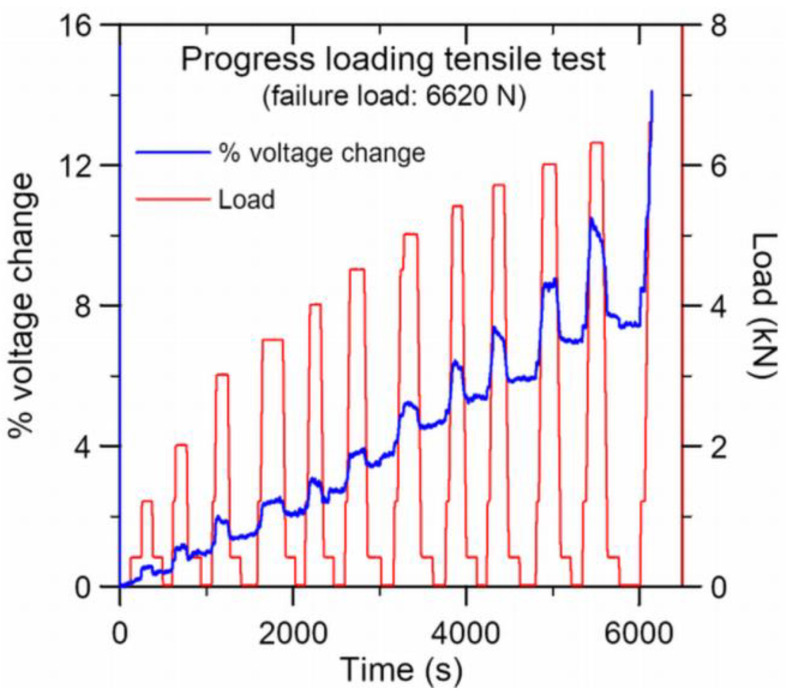
Percentage voltage change on progressive loading up and unloading.

The percentage voltage changes during the progressive loading up and unloading of the above specimen is shown in Figure 5. It is clear that not only does the voltage drop and thus the resistance of the lap joint specimen increases with load, but also that part of the increase is irreversible. The latter is evident from the percentage voltage change after unloading to 400 N and 0 N in each loading–unloading cycle. This trend was observed in 22 out of 25 tensile specimens. The remaining three specimens showed fluctuation within ±1% changes toward the end of the tests. Of those showing an increasing resistance with load, the load-free percentage change in voltage after unloading from 95% of the failure load varied widely from approximately 3% to 30%, with an average of 7%.

Application of tensile load to the lap-joint specimen will cause deformation of and possibly microscopic damages in the adhesive joint, thus breaking some of the CNT contacts and increasing the inter-tube distances. This will result in a rise in electrical resistance, exemplified by an increase in voltage across the joint where a constant current passes. On unloading, elastic deformation will be recovered, while plastic deformation will not. Internal defects may be partially or totally closed up when the deformation is recovered. Thus, the percentage voltage changes after each loading–unloading cycle may be attributed to the irreversible plastic deformation and microscopic internal defects that remained open.

**Figure 6:**
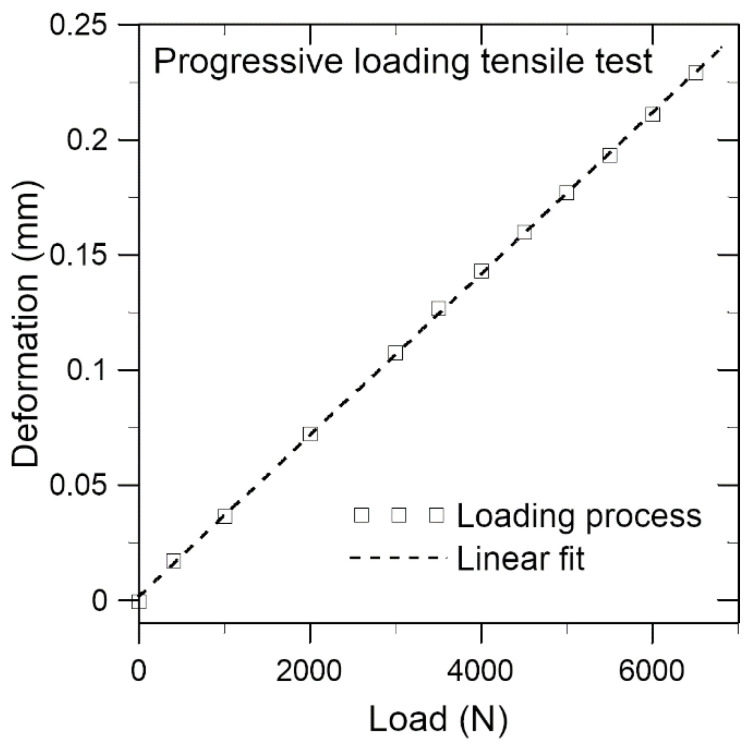
Specimen elongation versus maximum load for each progressive loading cycle.

A graph depicting the specimen elongation against the maximum load at each progressively loading-up cycle (Figure 6) shows that the data points virtually lie on a linear fit up to 98% of the failure load. This suggests that the deformation is essentially elastic. The corresponding peak shifts of the unloaded spectra of the three embedded FBGs, which are much more sensitive deformation sensors, corroborated with this induction. They recorded small permanent tensile strains of 40–60 με after various loading cycles in this specimen. From Figure 6, a deformation of 60 με, or 0.0015 mm out of the 25 mm long adhesive joint, corresponds to an applied load of approximately 40 N. Figure 5 shows that the voltage changes by 0.52% under 1200 N. Thus, a loading of 40 N, or deformation of 60 με, by itself should only lead to a negligibly small conductivity change. Therefore, the observed 7% voltage change after unloading from 95% of the failure load may be attributed to damages inside the joint.

**Figure 7:**
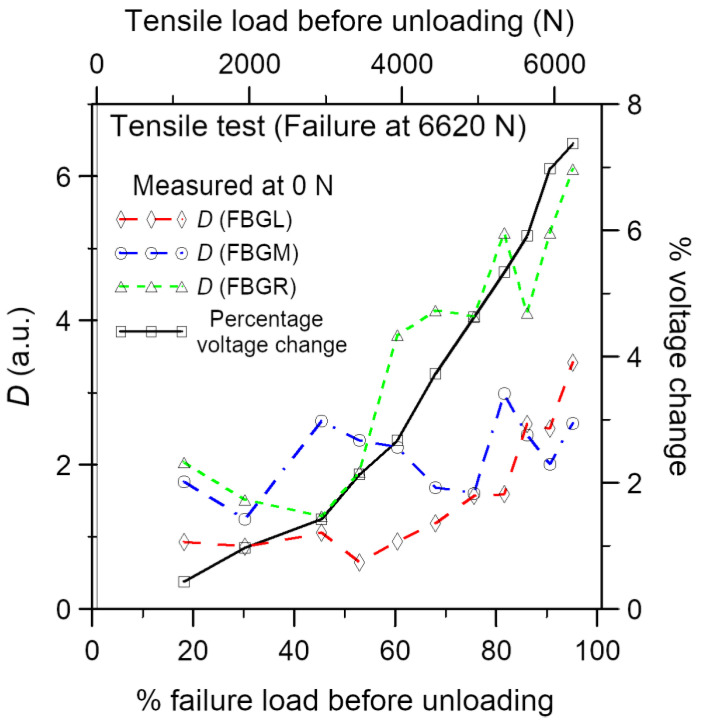
Load-free percentage voltage change and the three FBG spectra against the maximum load of the loading–unloading cycle.

To compare the results of damage sensing by the FBGs and conductivity changes, the distortion in the load-free FBG spectra after progressive loading has been quantified with the *D* value proposed above. Figure 7 shows these load-free *D* values and percentage voltage changes at zero load against the previous maximum load reached in the loading–unloading cycle. On progressively loading up, the percentage voltage change increases monotonically to approximately 7% near specimen failure. The *D* value for the FBGR spectra closely follows the same trend. The rising trend of the *D* values from FBGL starts somewhat later at approximately 50% of the failure load. That of FBGM fluctuates and shows no clearly increasing trend. The differences among the three FBGs may be caused by different amounts of localized damage near the corresponding FBG. The *D* values involved range from 1 to 6. As noted in Figure 4 above, these correspond to barely discernible changes in the FBG spectra, and the internal damages involved are believed to be small.

#### 3.1.3. Examination of Damage in the Progressively Loaded Tensile Specimens

**Figure 8:**
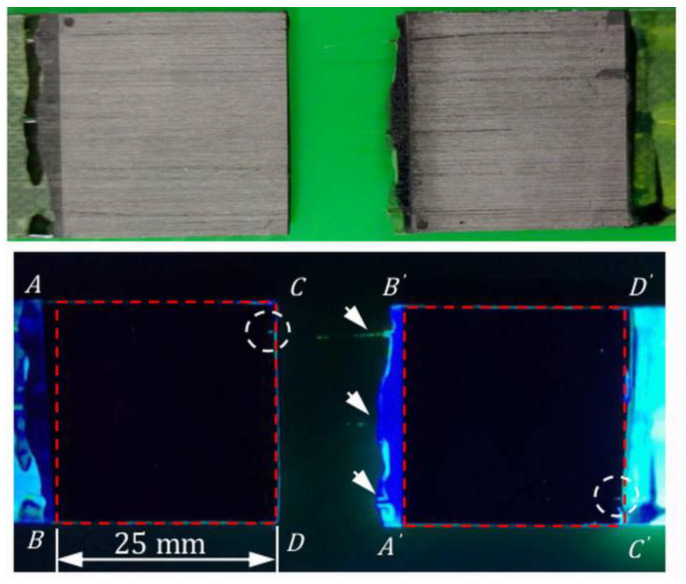
Fractured adhesive joint observed under visible light (**top**) and UV light (**bottom**) after fluorescent penetrant treatment.

In two of the progressively loaded tensile specimens, tests were interrupted after loading up to close to the failure load. The specimens were soaked in fluorescent penetrant, baked dry thoroughly, and pulled to failure. Figure 8 shows the fracture surfaces on the two halves of an adhesive joint specimen lying side by side under visible and ultraviolet light in a dark environment. This specimen was loaded to 5820 N (92% of the batch tensile strength) before soaking in the fluorescent penetrant. The maximum *D* values recorded among different FBGs was 7, and the percentage voltage change was 5% just before penetrant treatment. The red dotted squares in Figure 8 are the boundary of the joint. Alphabet pairs such as *A* & *A*’ represent the same points in the original lap joint specimen. The bright green color outside the joint boundary is the remnant of the dried penetrant. As pointed out above, maximum stress and deformation occur at both longitudinal edges *AB* and *CD*, which are the most likely place to find indications. Within the joint boundary, everywhere except inside the white dotted circle showed no fluorescent indication. Inside the white dotted circle, a straight-line indication approximately 1 mm in length can be seen on corresponding locations on both halves. The shape of this indication is not typical of micro-cracking or debonding failure. Incidentally, this is the location of one of the optical fibers (indicated with white arrows) and may be the result of a small lack of bonding under the optical fiber. The above observations show that at a load that is above 90% of the failure strength, the joint was still largely intact. Essentially, the same phenomena were observed in another similarly treated specimen. The above observation corroborates the phenomenon that the characteristic FBG waveform remained virtually unchanged in shape, even when close to the failure load. We may tentatively assume that *D* values below 7 and a percentage change of voltage within 5% are associated with insignificant internal damages and no debonding.

**Figure 9:**
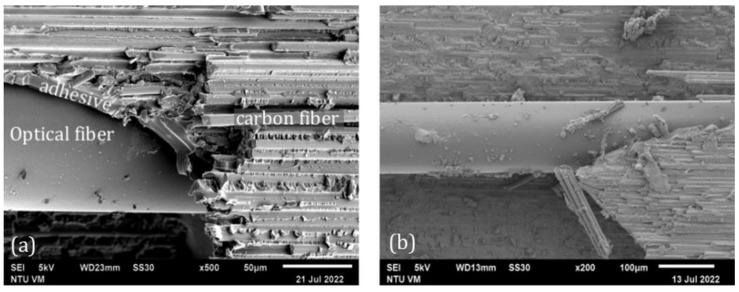
Typical SEM fractographs from two progressively loaded tensile specimens. (**a**) higher magnification; (**b**) lower magnification.

Typical scanning electron microscope (SEM) fractography for two different tensile specimens is shown in Figure 9. Dominating the fracture surfaces are carbon fibers, with epoxy resin between the fibers. Final failure clearly occurred in the carbon fiber epoxy composite adherent close to the adhesive–composite interface. Apart from occasional debris, the surface of the optical fibers is very smooth without any remnant adhesive attached. On the left micrograph, a thin layer of epoxy adhesive can be seen between the optical fiber and the composite adherent. This layer of adhesive appears to have been de-bonded from the optical fiber. Interfacial bonding between the optical fiber and the epoxy adhesive is probably relatively weaker than the adhesive, leading to cleavage at the optical fiber-adhesive interface during failure. This is in marked contrast to the SEM fractographs in our previous paper [46], which shows that the optical fiber is fully covered with adhesive after failure. Cracking and deformation of this attaching adhesive can be seen all over the optical fiber exterior. The reason for this difference may be that the CNTs added for conductivity purposes may also have reinforced the epoxy to make the adhesive stronger. The inferior adhesive strength to interfacial strength in Ref. [46] facilitated extensive micro-cracking and fracture in the adhesive, while the adhesive was still well bonded to the optical fiber. Localized strain concentration and perturbation due to internal damages in the vicinity of the optical fiber is easily transferred to the grating. This explains the early and significant changes in the unloaded FBG spectrum shapes after approximately 70% of failure strength in Ref. [46]. In the current case, the stronger reinforced adhesive probably limited its own micro-cracking and so the FBG spectra remained unchanged in shape, even beyond 90% of the failure load. Better adhesive strength also corroborates the lack of debonding fluorescent indication along the longitudinal edge.

### 3.2. Monitoring of Fatigue Damages

#### 3.2.1. FBG Spectra under Cyclic Fatigue Loading

**Figure 10:**
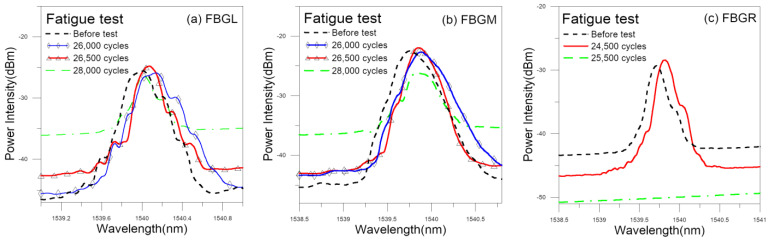
Load-free spectra measured in a typical fatigue specimen after various loading cycles.

**Figure 11:**
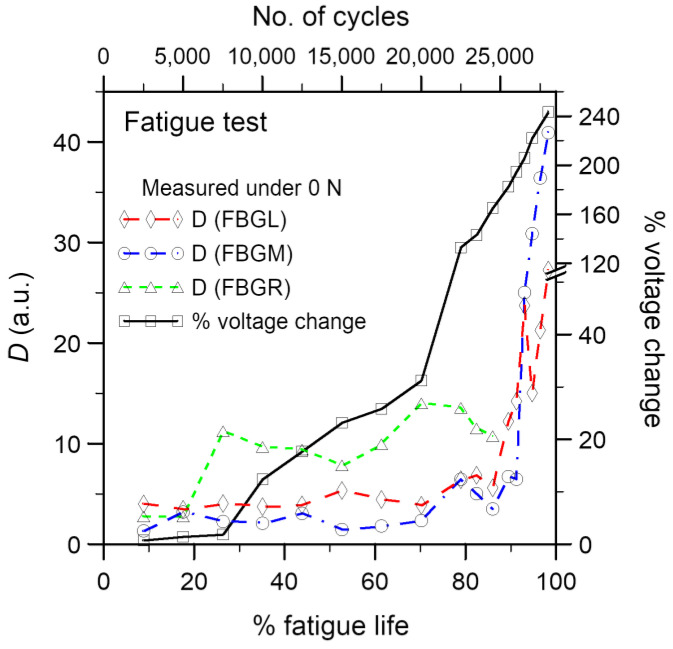
Load-free *D* values from three FBGs and the percentage voltage change development in a typical fatigue specimen.

A number of specimens have been subjected to cyclic loading, with the spectra of the embedded FBGs monitored periodically. Again, FBG spectra were recorded with the specimens unloaded to 400 N and 0 N. Figure 10 shows some of the spectra from a typical fatigue specimen. During the initial loading cycles, the waveform mainly shifts gradually to the right, with nearly no change in shape. When the specimen is getting close to final failure, marked changes in the waveform shape of the spectra start to occur. The starting point for this change varies with specimen, but in general it occurs after 80% of fatigue life has elapsed. Figure 11 shows the spectral shape changes quantified in *D* value. For FBGL and FBGM, the waveform shape starts to change significantly at 26,000 cycles, or 91% of fatigue life. Their *D* values remained below 5 before this and rose sharply afterward. For FBGR, the intensity of the spectrum increased at about 20% of fatigue life without significantly changing in the waveform shape. This persisted to 24,500 cycles, or 86% of life. The reflected peak disappeared in the next measurement. This makes the *D* value jumps to above 10 at the beginning and stays between 8 and 14 for the rest of life until the disappearance of the FBG spectrum at 25,500 cycles. It is not clear whether the above phenomena are due to a malfunction of the optical fiber or the effect of localized damage in the joint. The majority of FBGs (30 out of 35) behave in a similar way as FBGL and FBGM.

#### 3.2.2. Development of Percentage Voltage Change under Cyclic Fatigue Loading

Conductivity across the adhesive joint decreases with increasing loading cycle. In Figure 11, the percentage change in voltage has been shown alongside the *D* values from the FBGs in the same specimen. The *D* values remained more or less unchanged before 80% of fatigue life and rose steeply after that. On the other hand, there are roughly three stages of change in the voltage. The percentage voltage change remains low at approximately 1–2% before 26% of life. It rises gradually but significantly to 32% at 70% of fatigue life. More abrupt rising then occurred and reached 249% at 98% of life. It appears that the joint resistance sensed some progressive damages from 26% of life on. The FBG spectra gave no response, perhaps because such damages have not yet spread to their vicinity. Beyond 80% of life, damages spread to the FBGs and aggravated rapidly, causing steep rises in both the *D* values and the percentage voltage change.

**Figure 12:**
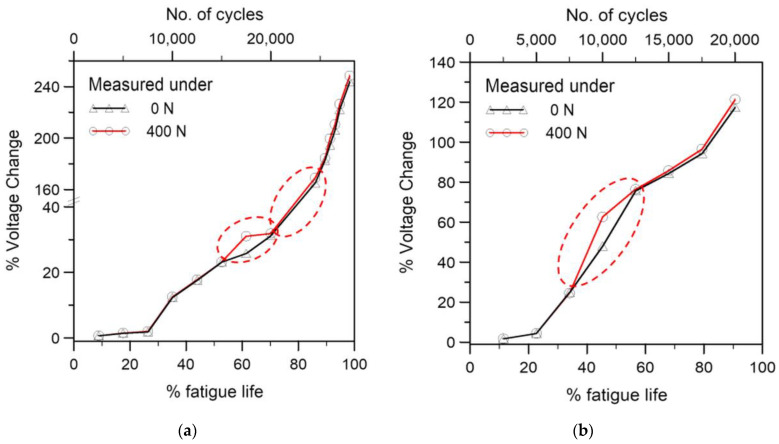
Comparison of percentage voltage change development measured at 0 N and 400 N in two fatigue specimens, note the difference at (**a**) 60% life; (**b**) 45% life.

A comparison of the percentage voltage changes after unloading to 400 N and 0 N may reveal some finer details about the nature of the joint damage. Figure 12 compares these for two fatigue specimens. Figure 12a is the same specimen as that in Figure 10 and Figure 11. The difference between the percentage changes in voltage under 400 N and 0 N are within a few tenths percent initially. This corroborates a previous observation that an applied loading of 1200 N changed the voltage by 0.52%, and so the difference between 400 N and 0 N are minimal. However, at some point in life, the 400 N reading is more than 2% higher than the 0 N reading (e.g., at 60% life in Figure 12a and 45% life in Figure 12b). As cyclic loading continues, their difference reverts to a much smaller value. This kind of phenomenon has been marked with red dotted ellipses in Figure 12. This probably occurred when part of the broken contacts/increased inter-tube distances in the CNT network due to fatigue damage was undone by recovery of elastic deformation by fully unloading but was held open by the 400 N load. On further damage development, this part of broken contacts/increased inter-tube distances became permanent, and the difference in voltage changes under 400 N and 0 N reverted to a fraction of a percent.

#### 3.2.3. Examination of Damages in the Fatigue Specimens

**Figure 13:**
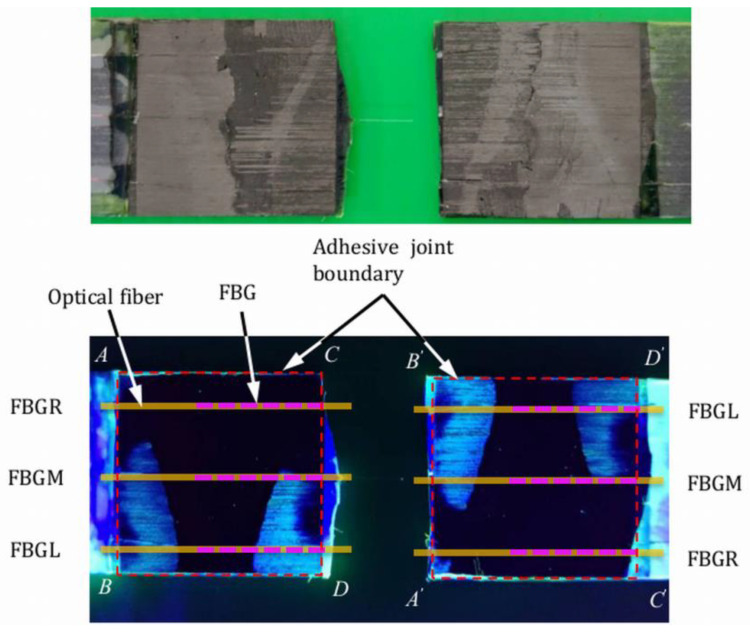
Fatigue-fractured adhesive joint observed under visible light (**top**) and UV light (**bottom**) after fluorescent penetrant treatment.

**Figure 14:**
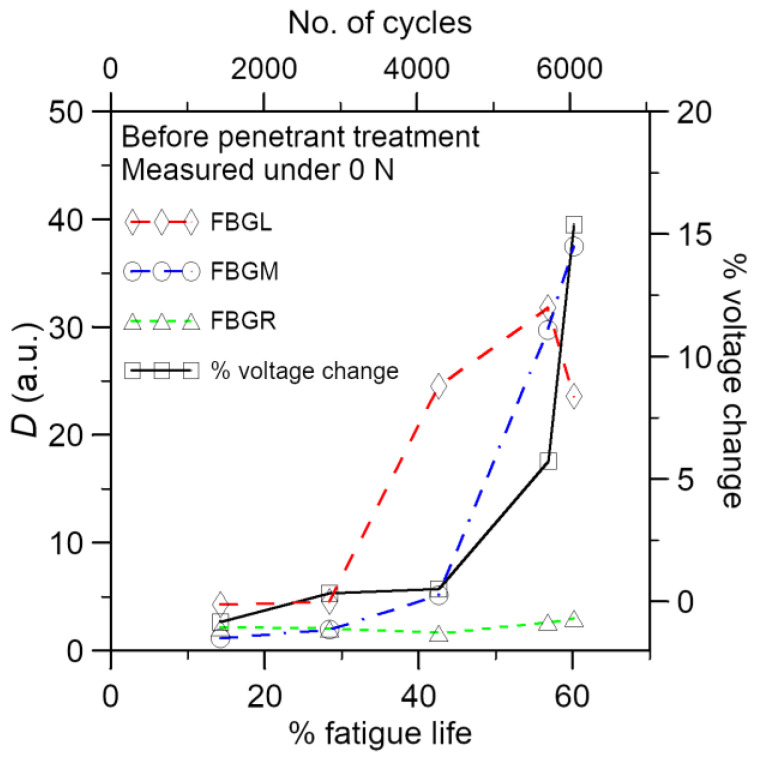
Load-free *D* values from 3 FBGs and the percentage voltage change development before the application of fluorescent penetrant in a fatigue specimen.

In some specimens, cyclic loading was interrupted when the percentage change in voltage reached either 10% or 15%. These specimens were then soaked in fluorescent penetrant and baked dry before cyclic loading was resumed until failure. Unlike that in the tensile tests, fluorescent indications on the adhesive joint can be seen in all fatigue specimens examined. Figure 13 shows a typical fractured joint under visible and UV light. Figure 14 shows the development of the percentage change in voltage and the *D* values in the same specimen. The percentage change in voltage reached was 15% just before penetrant treatment. Schematic representations of the optical fibers and relative locations of the FBGs (pink dotted lines) have been drawn on the joint in Figure 13. Clear fluorescent markings are evident under FBGL and FBGM. No indication is seen in the vicinity of FBGR. Incidentally, the *D* values from the FBGL and FBGM spectra rose to above 30 just before penetrant treatment (Figure 14). The shape of the spectrum from FBGR showed no significant change, and this was reflected in the *D* values, which remained below 4 throughout. It is worth pointing out that FBGR has not responded to the debonding approximately 4mm away under FBGM, suggesting that the sensing capability of FBG is highly localized.

**Figure 15:**
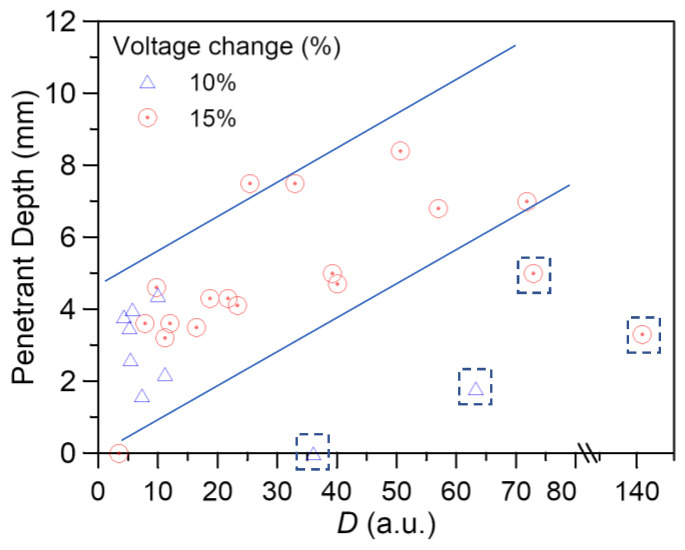
*D* values just before penetrant treatment versus the debonding length under or in the close vicinity of FBGs.

The depth of penetration of the fluorescent indications immediately under or within 1 mm vicinity of each FBG has been measured. These are plotted against the *D* values of the corresponding FBG spectra just before penetrant treatment in Figure 15. Apart from a few points (symbols boxed in dotted squares), the data fall roughly onto a scatter band, suggesting a strong correlation between the *D* value and the de-bonded depth. The boxed points that fall out of the scatter band are from the 5 FBGs with abnormal behavior in spectrum development, possibly due to malfunction. No debonding was evident in the penetrant examination of the tensile specimens, and *D* values remained below 7. These data from the tensile specimen fall into the same scatter band in Figure 15. Summarizing the above observations, it may be concluded that although the *D* value from the FBG spectrum is only loosely quantitative, it helps to relate the condition of debonding damage in the adhesive bond around the FBG in both the tensile and fatigue specimens.

**Figure 16:**
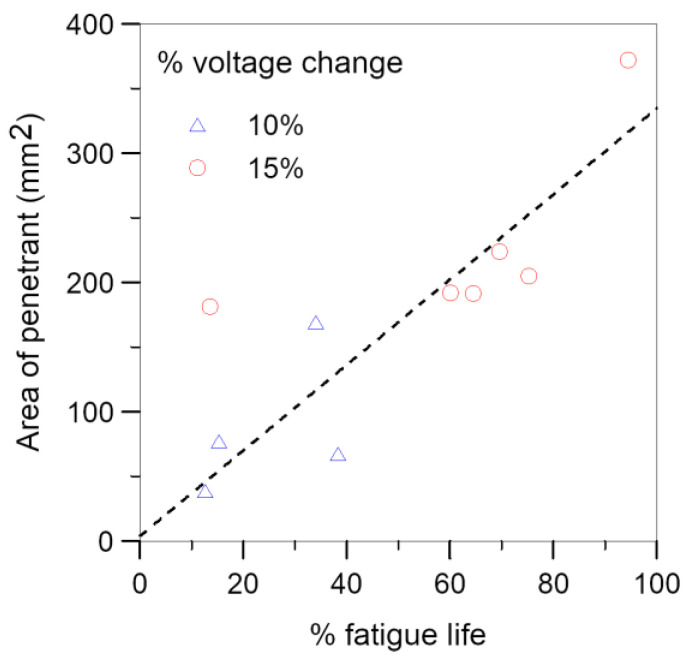
De-bonded area at different percentages of fatigue life.

Figure 16 shows the area of debonding against the fractional fatigue life spent just before penetrant treatment. Within experimental scatters, the fatigue life spent is roughly directly proportional to the de-bonded area. The data also indicated that the same percentage voltage changes could amount to widely different de-bonded areas and elapsed percentage fatigue lives. Thus, although the percentage voltage change helps to reflect the occurrence of debonding, it is not a good quantitative parameter to correlate directly with the de-bonded area or elapsed fatigue lives.

**Figure 17:**
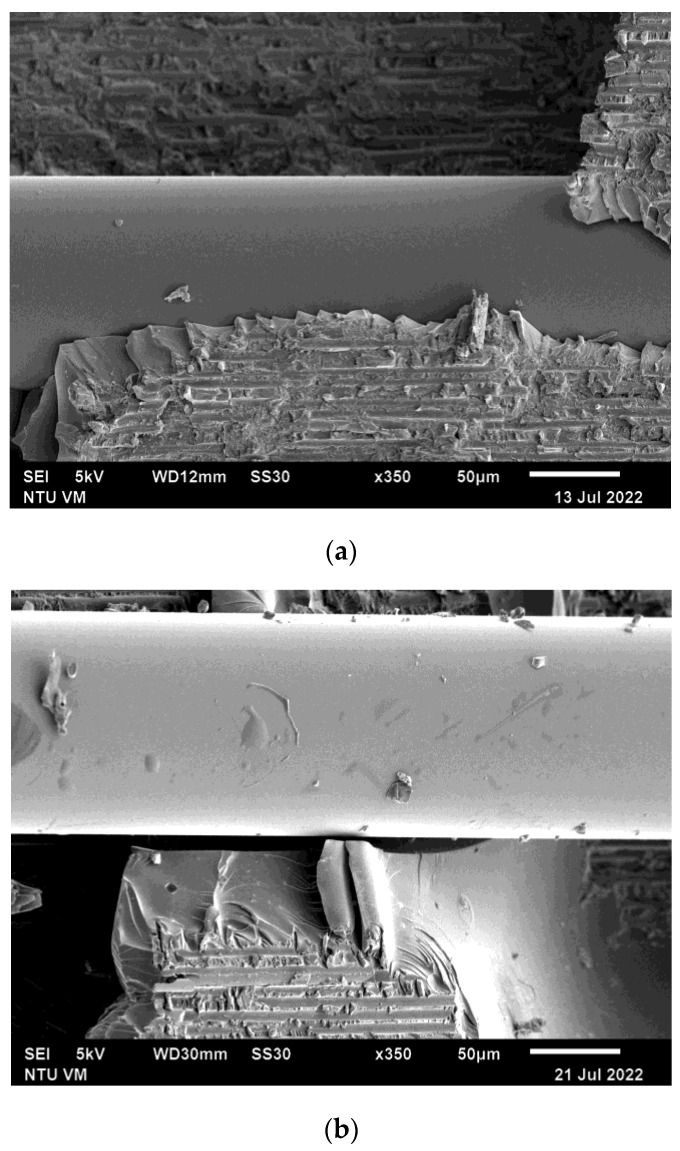
Typical SEM fractographs from two fatigue specimens showing (**a**) smooth optical fiber surface and (**b**) smooth optical fiber surface and secondary crack in adhesive.

Figure 17 shows typical SEM fatigue fractographs of the adhesive joint from two different fatigue specimens. Similar to the tensile failure cases, fractures mainly occurred in the composite lamina adjacent to the adhesive joint. The surface of the optical fibers appears to be very smooth and de-bonded from the adhesive. A secondary crack in the adhesive roughly perpendicular to the optical fiber can be seen in Figure 17b. This suggests that the spectral shape changes in the FBG spectra as well as the percentage voltage change throughout the cyclic loading may be contributed to by microcracking in the adhesive as well as debonding near the adhesive interface.

Summarizing the above observations, both the *D* value and percentage voltage change obtained under the load-free condition increase with applied tensile loading and elapsed fatigue life. They are more suited for monitoring failure mechanisms that involve a gradual formation and development of defects and are not so suitable for monitoring overload failure with little prior damage. A *D* value below 5 or a percentage voltage change below 5% may be used as an indicator of no debonding in the current joint specimens. The deviation of the percentage voltage change from 0, but below 5%, may be attributed to broken CNT contacts and increased inter-tube distances. The *D* value has a strong quantitative correlation with the debonding depth. However, its capability is highly localized and only reflects debonding in the close vicinity of the FBG, from which spectra the *D*’s are computed. On the other hand, the percentage voltage change responds to the bulk condition of the joint. It reflects joint degradation/damages and the occurrence of debonding qualitatively. Quantitatively, the same percentage voltage change is associated with widely different amounts of debonding and remaining fatigue lives, and so it is not a rigorous quantitative parameter to correlate fatigue lives or de-bonded areas directly. For practical structural health monitoring purposes, a specific value for the percentage voltage change, obtained through suitable experimental calibration, may be assigned as an alarm level. When this alarm value is reached, suitable action should be taken to prevent structural failure. For example, in the current fatigue specimens, a 10% voltage change may be set as an alarm level. It corresponds to readily detectable debonding, yet there is still a reasonable safety margin on fatigue life.

## 4. Conclusions

The integrity of CNT-doped adhesive single lap joint specimens under tensile and cyclic fatigue loading has been monitored with embedded FBG sensors and resistance of the joint specimens. A parameter *D* has been proposed to describe the change in FBG spectrum shapes quantitatively. The extent of joint debonding was revealed by fluorescent penetrant. The key findings can be summarized as follows:

(1) Loading by itself will affect the FBG spectrum and the voltage across a conductive adhesive joint, even in the absence of joint damage. Hence, for damage monitoring, the percentage change in voltage and the FBG spectrum should preferably be measured at zero or a very low load, in order to exclude the effect of load.
(2) The FBG spectrum and the associated *D* value only reflect the damages in the close vicinity of the FBG. The resistance or voltage drop across the joint under a constant current reflects the bulk condition of the joint.
(3) Under tensile loading, the load-free percentage change in voltage and *D* increased with progressively increasing loading. At a loading of 95% of failure strength, the average percentage change in voltage and the *D* value are 7% and 9%, respectively. Fluorescent penetrant testing showed that at 92% of failure strength, where the percentage change in voltage and *D* are 5% and 7%, respectively, no debonding in the joint had been observed.
(4) Under cyclic fatigue loading, the average change in voltage across the joint is 138%. This was measured at approximately 98% of fatigue life and was much larger than those recorded during tensile failure. *D* values from the FBG spectra varied widely, and most of them were also significantly larger than those recorded in tensile failure. Fluorescent penetrant tests revealed a strong correlation between the debonding depth in the vicinity of the FBG and their corresponding *D* value. However, the same percentage change in voltage may correspond to widely different de-bonded areas and elapsed fatigue life fractions.

## Data Availability

Data used for this study have been presented in the paper.
